# Polymorphism rs7079 in miR-31/-584 Binding Site in Angiotensinogen Gene Associates with Earlier Onset of Coronary Artery Disease in Central European Population

**DOI:** 10.3390/genes13111981

**Published:** 2022-10-30

**Authors:** Jan Novák, Soňa Maceková, Renata Héžová, Jan Máchal, Filip Zlámal, Ota Hlinomaz, Michal Rezek, Miroslav Souček, Anna Vašků, Ondřej Slabý, Julie Bienertová-Vašků

**Affiliations:** 1 Department of Pathological Physiology, Faculty of Medicine, Masaryk University, 625 00 Brno, Czech Republic; 2 Department of Physiology, Faculty of Medicine, Masaryk University, 625 00 Brno, Czech Republic; 3 Second Department of Internal Medicine, St. Anne’s University Hospital and Faculty of Medicine, Masaryk University, 656 91 Brno, Czech Republic; 4 Ondrej Slaby Joint Research Group, Central European Institute of Technology and Department of Biology, Faculty of Medicine, Masaryk University, Kamenice 5, 625 00 Brno, Czech Republic; 5 Department of Cardiovascular Diseases, St. Anne’s University Hospital and Faculty of Medicine, Masaryk University, 656 91 Brno, Czech Republic; 6 International Clinical Research Center, St Anne’s University Hospital, 656 91 Brno, Czech Republic

**Keywords:** rs7079, angiotensinogen, restenosis, age, coronary artery disease

## Abstract

Angiotensinogen (AGT) represents a key component of the renin–angiotensin–aldosterone system (RAAS). Polymorphisms in the 3′ untranslated region (3′UTR) of the AGT gene may alter miRNA binding and cause disbalance in the RAAS. Within this study, we evaluated the possible association of AGT +11525C/A (rs7079) with the clinical characteristics of patients with coronary artery diseases (CAD). Selective coronarography was performed in 652 consecutive CAD patients. Clinical characteristics of the patients, together with peripheral blood samples for DNA isolation, were collected. The genotyping of rs7079 polymorphism was performed with TaqMan^®^ SNP Genotyping Assays. We observed that patients with the CC genotype were referred for coronarography at a younger age compared to those with the AA+CA genotypes (CC vs. AA+CA: 59.1 ± 9.64 vs. 60.91 ± 9.5 (years), *p* = 0.045). Moreover, according to the logistic regression model, patients with the CC genotype presented more often with restenosis than those with the CA genotype (*p* = 0.0081). In conclusion, CC homozygotes for rs7079 present with CAD symptoms at a younger age compared with those with the AA+CA genotype, and they are more prone to present with restenosis compared with heterozygotes.

## 1. Introduction

The renin–angiotensin–aldosterone system (RAAS) is one of the most important systems involved in the regulation of blood pressure on the systemic level. The RAAS also affects other various processes, such as aging, fibrosis, or inflammation in its numerous local/tissue variants [1]. The central molecule of this system is angiotensinogen, an α2-globulin protein synthesized by the liver, which after N-terminal cleavage with renin gives rise to angiotensin I (AngI) and other derived angiotensin peptides that are either part of the “classical” RAAS pathway (e.g., AngII, AngIII) or to the “alternative” RAAS pathway (e.g., Ang1-9, Ang1-7) [1]. Maintaining balance between both RAAS branches is necessary for proper RAAS function, and while the increased activity of the classical pathway is known to be associated with the development of hypertension, coronary artery disease (CAD), and heart failure, the activation of an alternative pathway is considered cardioprotective [2].

Variability in the gene expression of individual RAAS genes and, consequently, the activity of individual RAAS proteins are known to be partially affected by their genetic background, as there is a plead of genetic polymorphisms that are scattered throughout individual RAAS genes (e.g., insertion–deletion polymorphism in angiotensin-converting enzymes (ACEs) affect ACE activity, which is increased in individuals with the DD genotype [3], and promoter polymorphism C-344T in aldosterone synthases is known to alter the binding of steroidogenic transcription factor 1 (SF-1), which results in increased aldosterone levels in individuals with the −344C genotype [4]). In general, individual polymorphisms can be located in the promoter regions, within intronic and exonic sequences, and also in 3′-untranslated regions (3′-UTR) [5]. With the discovery of microRNAs (miRNAs, miRs), which are tiny molecules that post-transcriptionally affect gene expression mostly by binding to the 3′UTRs of their target messenger RNAs (mRNAs) based on base pairing (C-G, A-T), polymorphisms in 3′-UTR have gained novel attention, as altered base pairing may alter miRNA binding and protein expression on post-transcriptional levels [5].

+11525C/A (rs7079) polymorphism is located within the 3′UTR of the angiotensinogen gene in the binding site of two distinct miRNAs, miR-31 and miR-584 [6,7]. These two miRNAs show fully complemental binding to the C variant of rs7079 polymorphisms, while they bind less to the A variant [6,8], which alters AGT expression and serum levels [8]. As the RAAS is known to be involved in the development of CAD, we hypothesized that rs7079 might alter the clinical course and future outcomes of CAD patients.

## 2. Materials and Methods

### 2.1. Study Population

The current study was a retrospective study using a previously prospectively enrolled cohort of stable coronary artery disease (CAD) patients that were indicated for coronary angiography at the Department of Cardiovascular Diseases, St. Anne’s University Hospital in Brno, Czech Republic. The Department of Cardiovascular diseases of St. Anne’s University Hospital in Brno is a highly specialized tertiary center offering expert cardiovascular care that covers the whole spectrum of cardiovascular diseases. Before enrollment into the study, all patients signed an informed consent form that was approved by the Ethical Committee of Masaryk University (No. MU-LF-154/06/2014), and the study was performed in line with the Declaration of Helsinki.

Patients were enrolled between the 1 January and 31 December 1998. Only patients indicated for coronary angiography due to angina pectoris without a laboratory finding of myocardial damage were enrolled; patients with acute coronary syndrome or with laboratory findings reflecting myocardial damage were not enrolled. In the mentioned timeframe, 810 consecutive Caucasian Central European patients were enrolled; for the current study, DNA samples were available for 652 cases.

Basic anthropometric and anamnestic parameters were collected, including age at the coronary angiography, weight, height, BMI, systolic and diastolic blood pressures, and pharmacological anamnesis. Within the standard operating procedures, echocardiography was performed to determine the ejection fraction of the left ventricle, and blood sampling was performed to determine basic laboratory parameters in the St. Anne’s University Hospital’s accredited laboratory (including serum levels of low- and high-density lipoproteins (LDL, HDL), triglycerides, and glucose).

For this study and according to our previous study [9], CAD was defined as the presence of at least 50% stenosis of any segment of any major coronary artery in two-dimensional imaging. If at least 50% stenosis was present in the coronary artery where a previous percutaneous coronary intervention was performed, it was classified as restenosis. All coronary angiography findings were evaluated by two independent, experienced cardiologists. Based on the number of affected main coronary arteries (i.e., the left anterior descendent branch (LAD), left circumflex branch (LCx), or right coronary artery (RCA)), one, two, or triple vessel diseases were defined. Further data, such as the total number of stenoses and their location within the individual coronary arteries, were also evaluated and documented.

Patients without any narrowing of the coronary arteries (*n* = 81) and patients with a severity of stenosis below 50%, as well as those who had a heart transplantation or were suspected vasospastic angina (*n* = 47), were excluded from the current study. After applying all the described criteria, 514 patients with CAD were included for further analysis in this study.

### 2.2. Remote Follow up after 15 Years

The signed informed consent form made it possible to further use and repeatedly access the clinical data and DNA samples of the patients for research purposes. Fifteen years after the enrollment of the last patients, the electronic information system of St. Anne’s University hospital, together with the data from The Institute of Health Information and Statistics of the Czech Republic, was used remotely (i.e., without contacting the enrolled subjects directly) to assess their all-cause mortality. Mortality data were obtained from 506 individuals and were used for the construction of Kaplan–Meir curves and the estimation of the patient’s survival.

### 2.3. Genotyping

From each patient, 5 mL of venous peripheral blood was collected into EDTA tubes and stored at −20 °C until DNA isolation. Genomic DNA was isolated using the standard proteinase-K technique.

The genotyping of the selected SNP (rs7079) was performed with TaqMan^®^ SNP Genotyping assays (catalog number: 4351379, assay ID: C____204370_10) according to the manufacturer’s protocol using the StepOne^®^ real-time PCR system (Thermo Fisher Scientific Inc., Waltham, MA, USA).

The validity of the results was confirmed by a second genotyping of 5% of randomly selected samples. Within this confirmatory genotyping, previously identified genotypes were confirmed in all cases (there was a full match between the primary and secondary sampling).

The genomic distribution did not show a deviation of the studied polymorphism from Hardy–Weinberg equilibrium (χ^2^ = 1.4171, *p* = 0.23).

### 2.4. Statistical Analysis

Statistical analyses were performed using the R-software package (Reston, VA, USA). The Shapiro–Wilk test was performed to assess normality. According to the results of the normality testing, further appropriate statistical tests were used, including Mann–Whitney or Student’s test, the Kruskal–Wallis test, and Pearson’s or Fisher’s correlation coefficient. A survival analysis was performed using Kaplan–Meir curves. Logistic regression modeling was performed to determine the relationships among age, genotype, and the presence of restenosis, and to determine the effect of the studied polymorphisms on mortality.

## 3. Results

### 3.1. Basic Description of the Study Group

Altogether, 514 consecutive CAD patients (84 women) were enrolled in the study (mean age 60.1 ± 9.6 years, BMI 27.6 ± 3.2 kg/m^2^). The study group characteristics are provided in Table 1.

### 3.2. Comparison of Individual Genotypes

There were no statistically significant differences between the individual genotypes (CC vs. CA vs. AA) (Supplementary Table S1). We thus further created the dominant and recessive models comparing either CC+CA vs. AA or AA+CA vs. CC and observed that individuals with the CC genotype were referred for coronary angiography at a younger age compared with those with AA+CA genotypes (CC vs. AA+CA: 59.1 ± 9.64 vs. 60.91 ± 9.5 (years), *p* = 0.045). The individual genotypes did not differ in other basic descriptive parameters (Supplementary Table S2).

### 3.3. Correlation of AGT Genotypes with Categorical Variables

A statistically significant association was observed between the AGT polymorphism and the presence of restenosis (Fisher’s test, *p* = 0.0081). There was no statistically significant association between the AGT genotypes and sex, the total number of stenoses, the localization of stenoses, or the use of statin or fibrates.

### 3.4. Logistic Regression Modeling Focused on Restenosis Occurrence

**Figure 1:**
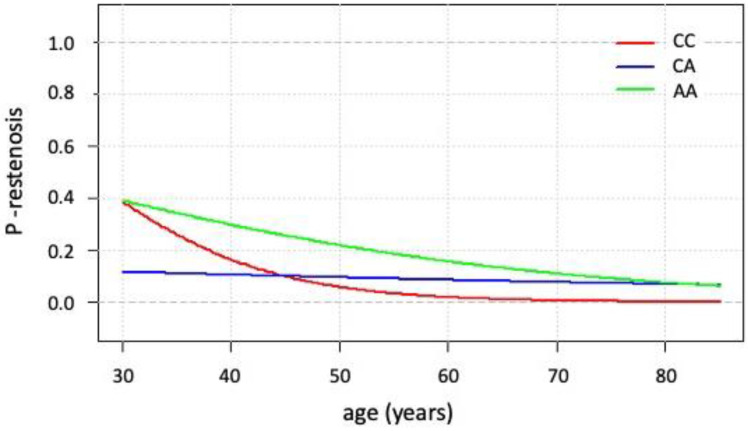
Logistic regression modeling estimating the presence of restenosis (dependent variable) for individual genotypes and ages (independent variables). A more detailed explanation can be found in the text.

Altogether, 34 patients with CAD presented in the study period with restenosis (6.6% of the whole CAD cohort). Out of these, 33 were men and 1 was a woman; thus, the female sex was excluded from further modeling. Due to the statistically significant differences in age among the individual genotypes at the time of the coronary angiography referral, age was included in the logistic regression modeling. Therefore, the occurrence of restenosis was considered a dependent variable, and AGT genotypes and age as independent variables. Using the backward stepwise regression method, we obtained individual models for each genotypes as follows: for the CC genotype, logit(P(restenosis = 1)) = 3.0378 − 0.1165 × age; for the CA genotype, logit(P(restenosis = 1)) = −1.6733 − 0.0116 × age; and for the AA genotype, logit(P(restenosis = 1)) = 0.8007 − 0.0414 × age. The final models are visualized in Figure 1. Significant differences were observed between the coefficients of the models between the CC and CA genotypes.

The interpretation of the model is as follows: in individuals with the CA genotype, the risk of restenosis remains almost the same at any age. In contrast, in individuals with the CC genotype and AA genotype, the risk decreases over time. The risk of restenosis is about ten times higher in younger individuals with the CC genotype compared with those with the CA genotype, while at the age of 45, this difference diminishes. Differences in restenosis occurrence between those with the CC or CA genotype compared with the AA genotype was statistically insignificant.

### 3.5. Kaplan–Meier Survival Analysis and Cox Analysis

**Figure 2:**
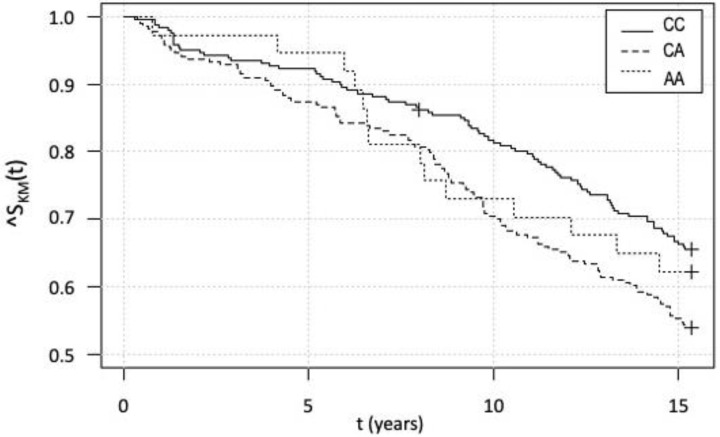
Kaplan–Meier survival analysis for individual genotypes within the 15-year-long follow up. A more detailed explanation can be found in the text.

We obtained 15-year all-cause mortality data for 506 individuals. In the follow-up period, 202 patients (40.9%) died and 304 patients (60.1%) survived. Data from the Kaplan–Meier analysis are shown in Figure 2.

**Figure 3:**
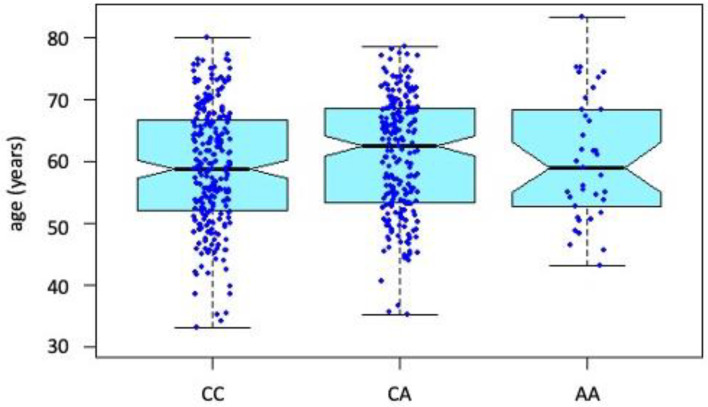
Distribution of age at the time of elective coronary angiography between individual genotypes.

At first glance, it may look like there is a difference in the survival function between the CC and CA genotypes. However, due to the above-described differences in age between the genotypes at the year of the selective coronarography, risk proportionality was verified using Cox proportional hazards modeling. As shown in Figure 3, individuals with the CA genotype are globally older than those with the other genotypes.

A Cox proportional hazards model for survival function (mortality) was thus created, taking age and AGT genotype as independent variables and showing that survival is statistically significantly affected only by age but not by the genotype.

## 4. Discussion

Single nucleotide polymorphisms (SNPs) are scattered through the genome and represent an eminent source of variability among individuals [5]. In the case of the RAAS, compelling evidence suggests that individual polymorphisms may affect the gene expression of individual RAAS molecules [3,4]. MicroRNAs represent post-transcriptional regulatory molecules whose action is functionally dependent on the base-pairing of A-T and C-G between the miRNA and its target mRNA, most commonly in its 3′UTR region [10]. Within our study, we focused on the SNP located within the 3′UTR of the AGT gene and observed that individuals with the CC genotype were referred to coronary angiography at a younger age. Furthermore, our analysis revealed that carriers of this genotype (especially men under the age of 45 years) were more prone to already present with the finding of restenosis, suggesting that their CAD started even sooner before the enrollment to our study. In our previous study, we showed that this polymorphism also affects fat distribution in the Central European population [7], and men with the C allele presented with a larger waist circumference, as abdominal obesity is a known risk factor for CAD development. Other authors showed that the rs7079C variant is also associated with the development of CAD and hypertension in the Saudi population [11]. In contrast, the rs7079A variant is more common in non-alcoholic steatohepatitis patients compared with control individuals in the Japanese population [12]. Altogether, these data suggest that rs7079 represents a functional polymorphism affecting the AGT expression and clinical characteristics of several patient groups.

Mechanistically, rs7079 is located within the 3′UTR of the AGT gene and affects the binding of two distinct miRNAs, miR-31 and miR-584 [6,8]. Standardized Luciferase assays showed that both miRNAs are fully compatible with the C allele, while they bind less to the A allele [6,8]. Intriguingly, in individuals with the CC genotype, serum levels of AGT were shown to be increased compared with individuals with the CA genotype [8]. We can hypothesize that increased levels of AGT in CC individuals may disrupt the delicate balance between the systemic and local RAAS, thus leading to faster atherosclerosis progression and resulting in CAD symptoms occurring at younger ages.

Furthermore, both miRNAs have already been shown to be involved in the regulation of blood pressure and in the pathophysiology of CAD, both of which are relevant to shed more light on the results of our study. Interestingly, both miRNAs were shown to be decreased in patients with CAD [13,14], and miR-31 levels specifically were decreased in the endothelial cells [13]. A decrease in the levels of both miRNAs together with altered binding caused by rs7079 polymorphism may hypothetically disrupt the balance between the systemic or local RAAS and affect CAD progression. Moreover, besides regulating AGT expression, both miRNAs were also shown to be involved in the regulation of nitric oxide production [15,16], which adds further levels of complexity to the potential effect of studied polymorphism on CAD development. Based on competitive endogenous RNA (ceRNA) theory [17], the increased binding of miR-31/584 to AGT in CC individuals may result in decreased amounts of the miR-31/584 available for nitric oxide synthase gene expression regulation. Additionally, miR-31 expression was shown to also be related to inflammatory processes, and low-level inflammation is known to be a part of the atherosclerosis process [18]. Levels of miR-31 are increased in endothelial cells under TNFα stimulation and affect the expression of E-selectin [19]. Furthermore, miR-31 affects the differentiation of Th22 lymphocytes [20], which are known to be pro-inflammatory and whose levels are also known to be increased in patients with acute forms of CAD [21].

Based on the results of the presented study, it seems that the processes underlying the effect of rs7079 on the earlier presentation of CAD in patients with the CC genotype may be multiple, which makes interpreting the data challenging and shall stimulate further research in this area.

## 5. Conclusions

In our study, we showed that rs7079 polymorphism CC homozygotes in the AGT gene are referred for coronary angiography at a younger age and are more prone to present with the finding of restenosis. The precise mechanisms need to be elucidated in further studies. Still, based on the literature search, this may be partially explained by the altered binding of miR-31/584 to the 3′UTR of the AGT gene and the disruption in RAAS balance.

## 6. Strengths and Limitations of the Study

The current study is especially limited by the low number of women in the study sample, which did not enable further statistical analysis and modeling of restenosis occurrence in females, which limits the generalization of the results. The study was also only monocentric, and the results can be applied only to the Central European population. Additionally, the absence of serum samples prevented us from measuring circulating levels of AGT and other vasoactive molecules (e.g., nitric oxide), which would be beneficial in explaining the effect of the studied polymorphism.

On the other hand, the study involved a large sample of patients with CAD, which was confirmed by up-to-date performed coronary angiographies, and utilized 15-year all-cause mortality follow-up data and provided a precise statistical evaluation and presentation of the data.

## Figures and Tables

**Table 1:** Study groups characteristics.

Parameter	
N (women)	514 (84)
Age (years)	60.10 ± 9.60
BMI (kg/m^2^)	27.57 ± 3.18
SBP (mmHg)	140.99 ± 18.65
DBP (mmHg)	83.92 ± 8.78
Cholesterol (mmol/L)	5.69 ± 1.10
HDL (mmol/L)	1.18 ± 0.33
LDL (mmol/L)	3.63 ± 1.05
Triglycerides (mmol/L)	2.11 ± 1.33
Glucose (mmol/L)	6.05 ± 1.70
LV EF (%)	50.37 ± 10.86

Abbreviations: CAD: coronary artery disease; N: number; BMI: body mass index; SBP: systolic blood pressure; DBP: diastolic blood pressure; HDL: high-density lipoprotein; LDL: low-density lipoprotein; LV EF: left ventricle ejection fraction; n.s.: non-significant.

## Data Availability

The source data are not available in publicly archived datasets and can be obtained from the authors upon formal request after obtaining the approval of the Ethical Committee.

## Supplementary Materials

The following supporting information can be downloaded at https://www.mdpi.com/article/10.3390/genes13111981/s1: Table S1: Comparison of individual genotypes within CAD group; Table S2: Dominant and recessive models of A and C alleles.
