# Supplementary material for: Polymorphism rs7079 in miR-31/-584 Binding Site in Angiotensinogen Gene Associates with Earlier Onset of Coronary Artery Disease in Central European Population
Source: Genes (Basel). 2022 Oct 30;13(11):1981. doi: 10.3390/genes13111981 (PMC9690213; doi:10.3390/genes13111981)
Supplement: Supplementary file 1 [file genes-13-01981-s001.zip › genes-1989727-supplementary.pdf]

genes

MDPI

Supplementary Tables

Supplementary Table S1: Comparison of individual genotypes within CAD group

AGT genotype:	CC	CA	AA	p- value
N	251	223	38	
**Age [years]**	59.1 ± 9.64	61 ± 9.43	60.31 ± 10.03	0.1120
**BMI [kg/m^2]**	27.61 ± 3.28	27.55 ± 2.98	27.56 ± 3.55	0.9900
**SBP [mmHg]**	141.15 ± 18.99	141.17 ± 18.5	138.18 ± 16.44	0.8078
**DBP [mmHg]**	83.89 ± 8.17	83.97 ± 9.57	83.84 ± 8.46	0.8581
**Cholesterol [mmol/l)]**	5.68 ± 1.11	5.72 ± 1.08	5.63 ± 1.24	0.8377
**HDL [mmol/l]**	1.18 ± 0.33	1.19 ± 0.34	1.12 ± 0.29	0.9380
**LDL [mmol/l]**	3.65 ± 1.07	3.64 ± 1.01	3.55 ± 1.26	0.8832
**Triglycerides [mmol/l]**	2.06 ± 1.31	2.18 ± 1.35	2.13 ± 1.4	0.3814
**Glucose [mmol/l]**	6.14 ± 1.92	5.97 ± 1.52	5.97 ± 1.17	0.9946
**LV EF [%]**	51.06 ± 10.65	50.09 ± 10.51	48.97 ± 13.55	0.4717

Abbreviations: CAD: coronary artery disease; N: number; BMI: body mass index; SBP: systolic blood pressure; DBP: diastolic blood pressure; HDL: high density lipoprotein; LDL: low density lipoprotein; LV EF: left ventricle ejection fraction; n.s.: non-significant

Supplementary Table S2: Dominant and recessive models of A and C allele

	CC+CA	AA	p-value	CA+AA	CC	p-value
N	474	38		261	251	
**Age [years]**	60 ± 9.58	60.31 ± 10.03	0.9685	60.91 ± 9.5	59.1 ± 9.64	**0.0454**
**BMI [kg/m²]**	27.58 ± 3.14	27.56 ± 3.55	0.9049	27.55 ± 3.07	27.61 ± 3.28	0.152
**SBP [mmHg]**	141.16 ± 18.75	138.18 ± 16.44	0.5511	140.73 ± 18.22	141.15 ± 18.99	0.9303
**DBP [mmHg]**	83.93 ± 8.84	83.84 ± 8.46	0.5854	83.95 ± 9.4	83.89 ± 8.17	0.9465
**Cholesterol [mmol/l]**	5.7 ± 1.09	5.63 ± 1.24	0.6716	5.71 ± 1.11	5.68 ± 1.11	0.7774
**HDL [mmol/l]**	1.18 ± 0.33	1.12 ± 0.29	0.7550	1.18 ± 0.33	1.18 ± 0.33	0.9673
**LDL [mmol/l]**	3.64 ± 1.04	3.55 ± 1.26	0.6179	3.63 ± 1.05	3.65 ± 1.07	0.9020
**Triglycerides [mmol/l]**	2.12 ± 1.33	2.13 ± 1.4	0.9438	2.17 ± 1.35	2.06 ± 1.31	0.1799
**Glucose [mmol/l]**	6.06 ± 1.74	5.97 ± 1.17	0.9190	5.97 ± 1.47	6.14 ± 1.92	0.9914
**LV EF [%]**	50.6 ± 10.58	48.97 ± 13.55	0.8444	49.92 ± 10.99	51.06 ± 10.65	0.2223

Abbreviations: N: number; BMI: body mass index; SBP: systolic blood pressure; DBP: diastolic blood pressure; HDL: high density lipoprotein; LDL: low density lipoprotein; LV EF: left ventricle ejection fraction; n.s.: non-significant.
